# Biomimetic Nanogels Programmed for Irreversible-Electroporation-Primed Tumor Microenvironments to Elicit Durable Antitumor Immunity

**DOI:** 10.34133/bmr.0359

**Published:** 2026-06-25

**Authors:** Jun-Hyeok Han, Ha Eun Shin, Chun Gwon Park, Hyun-Do Jung, Jung-Hoon Park, Ji Hoon Jeong, Yong Taik Lim, Dong-Hyun Kim, Wooram Park

**Affiliations:** ^1^ Department of Radiology, Feinberg School of Medicine, Northwestern University, Chicago, IL 60611, USA.; ^2^ Department of Integrative Biotechnology, College of Biotechnology and Bioengineering, Sungkyunkwan University (SKKU), Suwon, Gyeonggi 16419, Republic of Korea.; ^3^ Department of Biomedical Engineering, Institute for Cross-disciplinary Studies (ICS), SKKU, Suwon, Gyeonggi 16419, Republic of Korea.; ^4^ Department of Intelligent Precision Healthcare Convergence, ICS, SKKU, Suwon, Gyeonggi 16419, Republic of Korea.; ^5^ Division of Materials Science and Engineering, Hanyang University, Seoul 04763, Republic of Korea.; ^6^ Department of Convergence Medicine, Asan Medical Center, University of Ulsan College of Medicine, Seoul 05505, Republic of Korea.; ^7^ Biomedical Engineering Research Center, Asan Institute for Life Sciences, Asan Medical Center, Seoul 05505, Republic of Korea.; ^8^ School of Pharmacy, SKKU, Suwon, Gyeonggi 16419, Republic of Korea.; ^9^ Department of MetaBioHealth, School of Medicine, SKKU, Suwon, Gyeonggi 16419, Republic of Korea.; ^10^ Biomedical Institute for Convergence at SKKU, SKKU, Suwon, Gyeonggi 16419, Republic of Korea.; ^11^ SKKU Advanced Institute of Nanotechnology (SAINT), Department of Nano Science and Technology, Department of Nano Engineering, and School of Chemical Engineering, SKKU, Suwon, Gyeonggi 16419, Republic of Korea.; ^12^ Department of Biomedical Engineering, McCormick School of Engineering, Evanston, IL 60208, USA.; ^13^ Robert H. Lurie Comprehensive Cancer Center, Chicago, IL 60611, USA.

## Abstract

Irreversible electroporation (IRE) remodels the tumor microenvironment to enhance biomaterial and nanoparticle (NP) delivery and immune activation, making combinational IRE–nanomedicine a promising approach for effective cancer treatment. Here, we present a rational combination strategy that integrates IRE-induced immune modulation with M1 macrophage-membrane (M1-m)-coated nanogels to amplify and prolong antitumor immune responses. Transcriptomic and immunological profiling after IRE revealed a transient up-regulation of immune and inflammatory pathways, particularly the recruitment of macrophages and dendritic cells, followed by a rapid decline over time. To exploit this transient inflammatory state, we engineered an M1-m-coated nanogel hydrogel co-loaded with graphene quantum dots as a fluorescence probe and the immune modulator zoledronic acid (M1-GAZ). The IRE-enhanced tumor-targeting efficiency of M1-m-coated NPs was confirmed by comparing the tumor-targeting efficiency with other NP formulations including gold NPs (negatively or positively charged), lipid-based NPs (liposomes and lipid NPs, negatively or positively charged), and macrophage (M0 or M1) cell-membrane-coated NPs. Subsequently, the combination of IRE with intravenously injected M1-GAZ markedly increased the infiltration of activated macrophages and dendritic cells, resulting in superior tumor suppression and prolonged survival compared to monotherapies. This study demonstrates that engineering biomimetic M1-m-coated nanogels to synergize with IRE-induced tumor microenvironment remodeling enables selective delivery and durable immune activation, providing a robust platform for synergistic IRE cancer immunotherapy.

## Introduction

Cancer remains one of the leading causes of death worldwide, and despite advances in surgery, chemotherapy, and radiotherapy, the treatment of solid tumors still faces major challenges [1]. Conventional therapeutic modalities often fail to completely eradicate tumor cells and may inadvertently suppress systemic immunity, resulting in tumor recurrence and metastasis [2]. Therefore, strategies that not only ablate tumor tissues but also elicit potent and durable antitumor immune responses are critically needed to achieve long-term tumor control [3,4]. Among emerging local tumor ablation techniques, irreversible electroporation (IRE) has attracted increasing attention as a nonthermal approach that selectively destroys tumor cells while preserving the extracellular matrix and surrounding vasculature [5,6]. By applying high-voltage electrical pulses, IRE induces irreversible disruption of tumor cell membranes, leading to cell death without thermal injury [7]. This unique mechanism not only minimizes collateral damage but also facilitates immune cell infiltration and tissue remodeling in the treated region [8,9].

Recent studies have revealed that IRE can trigger immunogenic cell death and reshape the tumor microenvironment (TME) [10–12]. The release of tumor-associated antigens and danger-associated molecular patterns promotes the recruitment and activation of innate immune cells such as macrophages and dendritic cells (DCs) [13]. However, this immune activation is often transient and insufficient to establish systemic, long-term antitumor memory, representing a key therapeutic hurdle [3,9]. Furthermore, the therapeutic window created by IRE, characterized by enhanced vascular permeability and immune cell infiltration, is short-lived, limiting its potential for combination with systemic therapies [14,15]. In our previous and related studies, intratumoral delivery was effectively used to maximize local drug exposure during this window [11,12,16,17]. However, such approaches remain highly dependent on procedure and may limit broader clinical applicability, particularly in deep-seated or anatomically constrained tumors, in multifocal disease settings, or in scenarios requiring repeated dosing. Building on these experiences, leveraging this microenvironmental remodeling for efficient drug or nanoparticle (NP) delivery could therefore greatly enhance the therapeutic efficacy of IRE-based cancer treatments [14,15,18].

NP-based drug delivery systems offer several advantages for combination cancer therapy, including improved pharmacokinetics, targeted accumulation, and the ability to codeliver multiple therapeutic agents [19]. However, the therapeutic efficacy of certain small-molecule drugs, including zoledronic acid (ZOL), is often limited by rapid systemic clearance and poor tumor accumulation [20]. Moreover, simply coadministering free drugs with NPs does not necessarily reproduce the pharmacokinetic and tumor-retention advantages achieved through NP encapsulation [21]. Accordingly, rationally designed biomimetic NPs coated with natural cell membranes have been engineered to mimic biological interfaces, providing immune evasion, prolonged circulation, and precise therapeutic delivery [22,23]. Compared with other cell membrane coatings such as those derived from red blood cells, platelets, or cancer cells, macrophage membranes offer distinct advantages for tumor-targeted delivery [24–26]. Red blood cell and platelet membranes primarily confer prolonged circulation and immune evasion but lack active homing or inflammatory responsiveness [22]. Cancer cell membranes enable homologous targeting to tumors via self-recognition molecules but raise biosafety concerns due to potential oncogenic residues [27–29]. In contrast, macrophage-membrane-coated NPs inherit an inherent tropism toward inflammatory and necrotic tissues, mimicking the natural homing ability of macrophages [24,30,31]. This targeting behavior is mediated by adhesion molecules and chemokine receptors (e.g., CCR2, CXCR4, α4β1, and αMβ2) on the macrophage membrane that recognize endothelial adhesion molecules (e.g., intercellular adhesion molecule-1, vascular cell adhesion molecule-1, and E-selectin) up-regulated at inflammatory sites [30,32]. The membrane also displays self-markers such as CD47, which reduce phagocytic clearance and prolong circulation [33]. These features collectively enable selective accumulation of macrophage-membrane-coated NPs within IRE-treated tumors, where vascular permeability and immune recruitment are transiently enhanced.

To harness this potential, we developed an M1 macrophage-membrane-coated alginate nanogel (M1-GAZ) co-loaded with ZOL and graphene quantum dots (GQDs) for synergistic therapy with IRE. The alginate-based nanogel provides a biocompatible and injectable matrix capable of co-encapsulating multiple components, while ZOL serves as a potent immunomodulating agent known to repolarize M2-like macrophages toward a pro-inflammatory M1 phenotype and inhibit tumor progression. GQDs confer fluorescent traceability and potential photophysical activity for in vivo monitoring. We hypothesized that IRE would create a favorable microenvironment for M1-GAZ delivery, promoting selective tumor accumulation and amplifying immune activation.

In this study, we systematically investigated the biodistribution, therapeutic efficacy, and immune responses of the IRE and M1-GAZ combination in CT26-tumor-bearing mice. Our findings demonstrate that IRE-induced microenvironmental remodeling facilitates the selective accumulation of macrophage-mimetic nanogels and enhances antitumor immunity, providing a safe and effective approach for localized synergistic cancer therapy.

## Materials and Methods

### Materials

The following materials were used in this study: sodium alginate (Sigma-Aldrich), aluminum chloride (AlCl_3_; Sigma-Aldrich), chloroauric acid (HAuCl_4_; Sigma-Aldrich 254169), sodium citrate tribasic (Sigma-Aldrich S4641), hydroquinone (Sigma-Aldrich H17902), polyethyleneimine (PEI, branched, 25 kDa; Sigma-Aldrich 408727), 1,2-dioleoyl-sn-glycero-3-phosphoethanolamine (DOPE; Avanti 76548), 1,2-distearoyl-sn-glycero-3-phosphoethanolamine-*N*-[methoxy(polyethylene glycol)-2000] (PEG-PE; Avanti 880120P), 1,2-dioleoyl-3-trimethylammonium-propane (DOTAP; Avanti 890895P), dilinoleylmethyl-4-dimethylaminobutyrate (MC3; Avanti), 1,2-distearoyl-sn-glycero-3-phosphocholine (DSPC; Avanti), cholesterol (Avanti), 1,2-dimyristoyl-rac-glycero-3-methoxypolyethylene glycol-2000 (PEG-DMG; Avanti), ZOL (Sigma-Aldrich), pyrene (Sigma-Aldrich), nitric acid (HNO_3_; Daejung Chemicals), lipopolysaccharide (Sigma-Aldrich L2630), HEPES buffer (Thermo Fisher Scientific, 15630-056), phosphate-buffered saline (PBS; Gibco), and DiR fluorescent dye (DiIC_18_(7), D12731; Thermo Fisher Scientific).

### In vivo experiments

Eight-week-old female BALB/c mice were used for animal experiments after a 1-week acclimatization period. To establish a mouse tumor model, CT26 cells (2 × 10^6^ cells) were subcutaneously inoculated in the right flank of 9-week-old BALB/c nude mice. After 8 d, when the average volume of tumor reached 100 mm^3^, mice were used for the experiment (*n* = 3 mice per group). After mice were anesthetized entirely with isoflurane, IRE (voltage: 1,000 V, pulse duration: 100 μs, and electrode spacing: 5 mm) was applied to mice. Tumor volumes were calculated with the following formula: width × width × length × 0.5. Twenty-eight days after tumor inoculation, mice were sacrificed with CO_2_ gas when the tumor size exceeded 1,500 mm^3^.

### In vivo analysis of the expression of protein after IRE

IRE was applied using 8 pulses, 10 times, on CT26-tumor-bearing BALB/c mice at 8 d postinoculation under specific electrical field conditions (voltage: 1,000 V, pulse duration: 100 μs, and electrode spacing: 5 mm). Tumor tissues were collected at 0 h, 3 h, 6 h, 12 h, 1 d, and 3 d posttreatment for analysis (tumor samples from 3 mice per group were pooled prior to analysis). Protein expression profiles were assessed using the Proteome Profiler Mouse Apoptosis Array Kit (R&D Systems, Catalog #ARY031) according to the manufacturer’s instructions.

### In vivo analysis of tumor-infiltrating immune cells after IRE

IRE was performed on CT26-tumor-bearing BALB/c mice at 8 d postinoculation using 8 pulses repeated 10 times under specific electrical field conditions (voltage: 1,000 V, pulse duration: 100 μs, and electrode spacing: 5 mm). Tumors were harvested 3 d posttreatment (*n* = 3 mice per group). The harvested tumor tissue was digested into single cells as described above and then stained with the following antibodies: allophycocyanin (APC)-conjugated anti-CD45, PE-conjugated anti-F4/80 for macrophages, PE-conjugated anti-CD11c for DCs, fluorescein isothiocyanate (FITC)-conjugated anti-CD3 and PE-conjugated anti-CD335 for natural killer cells, and APC-conjugated anti-CD45 and PE-conjugated anti-Ly-6G for neutrophils. After staining for 30 min, the cells were washed and analyzed using an MA900 Multi-Application Cell Sorter (Sony Biotechnology, San Jose, CA, USA) (NFEC-2024-10-300262), and data were acquired and processed using flow cytometry software.

### Transcriptomic analysis after IRE

IRE treatment was performed on CT26-tumor-bearing BALB/c mice 8 d after tumor inoculation. Mice received 8 electrical pulses, repeated 10 times, under the following conditions: voltage, 1,000 V; pulse duration, 100 μs; and electrode spacing, 5 mm. Tumor tissues were harvested at 3 and 9 d after IRE treatment for transcriptomic analysis (tumor samples from 3 mice per group were pooled prior to analysis). Total RNA was extracted from tumor samples using standard procedures and analyzed with the Mouse nCounter Immuno-Oncology 360 Gene Expression Panel (NanoString Technologies), performed by PhileKorea Technology (Seoul, Republic of Korea) according to the manufacturer’s protocol. Data were normalized and analyzed using NanoString nSolver software to evaluate immune-related gene expression changes following IRE.

### Preparation of Au NPs

Gold (Au) NPs were synthesized using a citrate and hydroquinone reduction method as previously described [34]. Briefly, 0.962 ml of 25 mM chloroauric acid (Sigma-Aldrich 254169), 3.71 ml of 2.23 nM 15 nm seed Au NPs, 0.962 ml of 15 mM sodium citrate tribasic (Sigma-Aldrich S4641), and 0.962 ml of 25 mM hydroquinone (Sigma-Aldrich H17902) were added to 94.33 ml of deionized water and stirred overnight at room temperature. The resulting 50-nm Au NPs were characterized by dynamic light scattering (DLS). For cationic surface modification, the NPs were centrifuged at 1,500×*g* for 2 h, washed, and resuspended in 0.02% (w/v) sodium citrate solution. PEI was then added to achieve a final concentration of 0.1 mg/ml, followed by gentle stirring for 4 h at room temperature to allow electrostatic adsorption onto the gold surface. Excess PEI was removed by centrifugation (1,500×*g* for 30 min, performed 3 times), and the PEI-coated Au NPs were stored at 4 °C until further use.

### Preparation of liposomes

Liposomes were prepared following a previously reported method with slight modifications [16]. The lipids used were DOPE (Avanti 76548), 1,2-distearoyl-sn-glycero-3-phosphoethanolamine-*N*-[methoxy(polyethylene glycol)-2000] (18:0 PEG2000/PE, PEG-PE, ammonium salt, 880120P; Avanti), and DOTAP (Avanti 890895P). Lipids in powder form were dissolved in chloroform at a stock concentration of 1 mg/ml. Negatively charged liposomes were composed of DOPE/PEG-PE (1:1 molar ratio), and positively charged liposomes were composed of DOPE/PEG-PE/DOTAP (1:1:1 molar ratio). For in vivo distribution studies, 0.1 mol% of DiIC_18_(7) (D12731; Thermo Fisher Scientific) was incorporated into the lipid mixture. The lipid solution was evaporated at 60 °C and 200 rpm for 30 min using the thin-film hydration method to form a uniform lipid film. The dried lipid film was hydrated at 37 °C for 1 h with 20 mM HEPES buffer (Thermo Fisher Scientific, 15630-056) at a total lipid concentration of 2 mg/ml. Finally, the liposome suspension was extruded 11 times through a 200-nm polycarbonate membrane (Avanti Polar Lipids) to obtain uniformly sized vesicles. The resulting liposomes were stored at 4 °C until further use.

### Preparation of lipid NPs

Lipid NPs (LNPs) were prepared based on a previously reported method with slight modifications [35]. Lipid components (MC3, DSPC, cholesterol, and PEG-DMG) were individually dissolved in ethanol to prepare stock solutions (25 mM for MC3, DSPC, and cholesterol; 12.5 mM for PEG-DMG) and equilibrated at 37 °C for 10 min before mixing. For negatively charged LNPs, the 4 lipids were mixed at a molar ratio of 50:10:38.5:1.5 (MC3/DSPC/cholesterol/PEG-DMG). For positively charged LNPs, DOTAP (25 mM in ethanol) was incorporated to constitute 30 mol% of the total lipid composition. The lipid phase was mixed with an aqueous phase containing messenger RNA (mRNA) dissolved in 25 mM sodium acetate buffer (pH 5.2) at a volume ratio of 1:3 (lipid to mRNA) and a mass ratio of 1:20 (mRNA to total lipids) under gentle stirring. The resulting LNP suspension was dialyzed against PBS (pH 7.4) for 1 h to remove ethanol and neutralize the pH prior to use. For in vivo distribution studies, 0.1 mol% of DiIC_18_(7) (D12731; Thermo Fisher Scientific) was incorporated into the preformed LNPs by incubation at room temperature for 30 min. The DiR-labeled LNPs were then purified using an Amicon Ultra-10K centrifugal filter (Millipore) to remove unbound dye and residual solvent. The resulting LNPs were stored at 4 °C until further use.

### Preparation of M1 macrophage-derived NPs

Macrophages and macrophage-derived NPs were prepared following a previously reported method [31]. RAW264.7 murine macrophage cells were used to prepare both nonactivated (M0) and M1-polarized macrophage-derived NPs. Cells were cultured in Dulbecco’s modified Eagle medium (Gibco) supplemented with 10% fetal bovine serum and 1% penicillin–streptomycin at 37 °C in a humidified atmosphere containing 5% CO_2_. For M1 polarization, RAW264.7 cells were treated with lipopolysaccharide (1 μg/ml) for 24 h to induce a pro-inflammatory M1 phenotype. After harvesting cells, both M0 and M1 macrophages were washed 3 times with cold PBS and resuspended in PBS. Each cell suspension was continuously extruded through a polycarbonate membrane filter (Whatman, UK) with pore sizes of 10, 5, 1, 0.4, and 0.2 μm using a mini-extruder (Avanti Polar Lipids, AL, USA) to obtain M0- and M1-derived NPs (M0 NPs and M1 NPs). The concentration of M0 NPs and M1 NPs was determined using a bicinchoninic acid protein assay kit (Thermo Fisher Scientific), following the manufacturer’s instructions. For in vivo distribution studies, the NPs were labeled with DiIC_18_(7) (D12731; Thermo Fisher Scientific) by incubation at room temperature for 30 min, followed by purification using an Amicon Ultra-10K centrifugal filter (Millipore) to remove unbound dye. The purified DiR-labeled M0 NPs and M1 NPs were stored at 4 °C until further use.

### Preparation of M1-GAZ nanogels

GQDs were synthesized following a previously reported method [36]. Briefly, 2 g of pyrene was refluxed in 160 ml of concentrated HNO_2_ at 80 °C for 12 h under stirring. The resulting mixture was diluted with 1 l of deionized water and filtered through a 0.22-μm membrane. The obtained 1,3,6-trinitropyrene (3 g) was dispersed in NaOH solution and sonicated for 2 h, followed by hydrothermal treatment in a Teflon-lined autoclave at 200 °C for 10 h. The final product was filtered through a 0.22-μm membrane and dialyzed (molecular weight cutoff 3,500 Da) against deionized water for 2 d to obtain purified GQDs. To prepare M1-GAZ nanogels, 1 ml of alginate solution (10 mg/ml) was mixed with 250 μl of ZOL (10 mg/ml) and 250 μl of GQD solution (40 mg/ml), followed by the addition of 1 ml of AlCl_3_ solution (10 mg/ml) under gentle stirring. The resulting GQD/ZOL/Al mixture was then combined with 500 μl of M1-derived extracellular vesicles (10^10^ to 10^12^ particles per ml) and 500 μl of the GQD/ZOL/Al hydrogel precursor. The mixture was subsequently extruded 11 times through a 200-nm polycarbonate membrane (Avanti Polar Lipids) to form uniform M1-GAZ nanogels. Finally, the obtained nanogels were centrifuged at 10,000 rpm for 10 min at room temperature to remove unreacted components, and the purified M1-GAZ nanogels were collected for further experiments.

### DLS analysis

DLS measurements were performed to determine the hydrodynamic sizes of Au NPs (negatively or positively charged), lipid-based NPs (liposomes or LNPs, negatively or positively charged), macrophage (M0 or M1) cell-membrane-derived NPs, and GAZ and M1-GAZ nanogels. Measurements were carried out using a Zetasizer Nano ZS system (Malvern Panalytical, UK) equipped with a 633-nm He–Ne laser at a fixed scattering angle of 173° (backscattering configuration). All measurements were conducted at 25 °C after equilibrating the samples for at least 2 min prior to data acquisition. Samples were dispersed in distilled water, unless otherwise specified, and diluted to an appropriate concentration to avoid multiple scattering effects (typically 0.05 to 0.2 mg/ml for NP formulations). Each sample was measured in disposable polystyrene cuvettes, and at least 3 independent measurements were performed per sample, with each measurement consisting of multiple runs automatically determined by the instrument software. Particle size distributions were analyzed based on intensity-weighted size distributions, volume-weighted size distributions, and number-weighted size distributions. Size distributions were reported over the full nanoscale range (1 to 10,000 nm). To assess the quality and reliability of the DLS measurements, normalized intensity autocorrelation functions (*g*^2^) were analyzed for all samples. Representative correlation functions, along with the corresponding intensity-, volume-, and number-weighted size distributions, are provided in the Supplementary Materials. The smooth decay profiles and single-exponential behavior of correlation functions confirm the monodispersity and colloidal stability of NP formulations.

### In vitro experiments

DC2.4 murine dendritic cells and RAW264.7 were cultured in Dulbecco’s modified Eagle medium (Gibco) supplemented with 10% fetal bovine serum and 1% penicillin–streptomycin at 37 °C in a humidified atmosphere containing 5% CO_2_. Cells were seeded in 96-well plates (1 × 10^4^ cells/well) and incubated overnight, followed by treatment with various concentrations of M1-GAZ (0, 1, 5, 10, 20, 30, 40, and 50 μg) for 24 h. Cell viability was measured using the Cell Counting Kit-8 (Dojindo) by adding 10 μl of reagent to each well (final volume, 100 μl), incubating for 2 h at 37 °C, and measuring the absorbance at 450 nm using a microplate reader. To analyze cytokine and chemokine secretion, DC2.4 and RAW264.7 cells were seeded in 6-well plates (3 × 10^5^ cells/well) and treated with M1-GAZ (50 μg) for 24 h. The cell-free supernatants were collected and clarified by centrifugation (1,000×*g* for 10 min), followed by analysis using the Proteome Profiler Mouse Cytokine Array Kit, Panel A (R&D Systems, ARY006) according to the manufacturer’s instructions. Chemiluminescence signals were visualized, and dot intensities were quantified using ImageJ software after background subtraction and normalization to the reference spots and untreated controls for each cell type.

### In vivo analysis of tumor-infiltrating immune cells after IRE and M1-GAZ treatment

CT26-tumor-bearing BALB/c mice were treated with IRE at 8 d postinoculation under the following electrical parameters: 1,000 V, pulse duration 100 μs, 8 pulses per cycle, repeated 10 times, and with an electrode spacing of 5 mm. Immediately after IRE treatment, mice received an intravenous injection of M1-GAZ nanogels. Tumor tissues were collected 3 d after treatment and processed into single-cell suspensions (*n* = 5 mice per group). The tumors were enzymatically digested and filtered through a 70-μm cell strainer to obtain single-cell populations. The isolated cells were stained with the following fluorophore-conjugated antibodies for flow cytometry: FITC–anti-CD11b, APC–anti-CD80, and PE–anti-CD86 for macrophages; PerCP-Cy5.5–anti-CD11c, PerCP-Cy7–anti-CD40, and FITC–anti-major histocompatibility complex class II (MHCII) for DCs.

### Statistical analysis

GraphPad Prism 8 software (GraphPad Software, San Diego, CA, USA) was used to conduct the statistical analysis. All data were evaluated as the means ± standard deviations or the means ± standard error of the mean. Analysis of variance was used to examine 3 or more groups, while the *t* test was used to study 2 groups. The Shapiro–Wilk test was used to determine the normality of the data. The figure legend indicates the significance level of the *P* values.

## Results and Discussion

### Time-dependent molecular and immune responses following IRE treatment

Before conducting mechanistic and therapeutic evaluations, we first optimized the IRE parameters to determine the most effective pulse condition. Based on transcriptomic profiling (Fig. S1), the 80-bipolar-pulse condition at 1,000 V induced the strongest up-regulation of immune- and stress-related genes 3 d after treatment, indicating efficient tumor ablation and immune activation. Therefore, all subsequent experiments were conducted using IRE (1,000 V and 80 bipolar pulses) as the standard condition. To ensure biosafety, comprehensive serum biochemical analyses were performed under the same parameters (Fig. S2). No significant alterations were observed in hepatic (aspartate aminotransferase, alanine aminotransferase, and alkaline phosphatase), renal (blood urea nitrogen and creatinine), or cardiac (creatine phosphokinase and lactate dehydrogenase) markers, confirming that the selected IRE condition caused no systemic toxicity or organ damage.

**Fig. 1.**
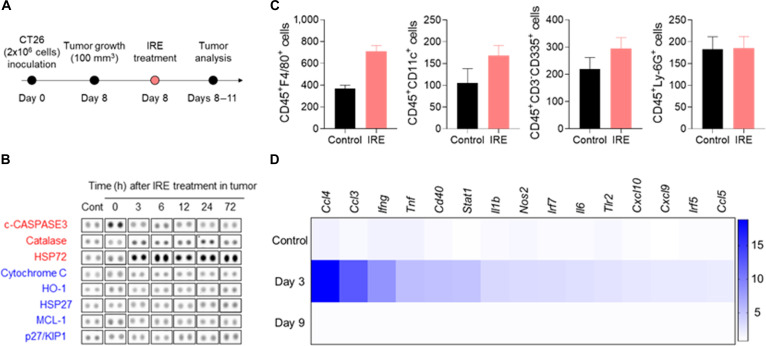
Time-dependent molecular and immunological responses following irreversible electroporation (IRE) treatment in a CT26 tumor mouse model. (A) Schematic representation of the in vivo experimental schedule. CT26 cells (2 × 10^6^) were subcutaneously inoculated into mice, and IRE was performed when tumors reached approximately 100 mm^3^. Tumors were collected for analysis at the indicated time point. (B) Temporal protein expression profiles in tumors after IRE treatment. Apoptosis- and stress-related proteins, including cleaved caspase 3 (c-CASPASE3), Catalase, heat shock protein 72 (HSP72), Cytochrome C, heme oxygenase-1 (HO-1), HSP27, myeloid cell leukemia-1 (MCL-1), and cyclin-dependent kinase inhibitor 1B (p27/KIP1). (C) Quantification of tumor-infiltrating immune cell populations 3 h after IRE treatment (*n* = 3). (D) Gene expression profiles of inflammation- and immune-related factors (e.g., Ccl4, Ccl3, Ifng, Tnf, Cd40, Stat1, Il1b, Nos2, Irf7, Il6, Tlr2, Cxcl10, Cxcl9, Irf5, and Ccl5) at 3 and 9 d post-IRE.

To investigate how IRE modulates the TME, we performed a time-dependent molecular and immunological profiling in CT26-tumor-bearing mice. Tumors were collected at 3 h, 1 d, 3 d, and 9 d after IRE treatment and analyzed for protein expression, immune cell composition, and transcriptional changes (Fig. 1A). Cytokine analysis revealed dynamic alterations in apoptosis- and stress-response-associated proteins (Fig. 1B). In particular, cleaved caspase-3, Catalase, and heat shock protein 72 (HSP72) were up-regulated during the early phase (3 to 6 h), whereas Cytochrome C, HSP27, myeloid cell leukemia-1, and cyclin-dependent kinase inhibitor 1B showed no noticeable change throughout the observation period. These results indicate that IRE simultaneously activates oxidative stress and apoptotic signaling pathways within the tumor tissue. Flow cytometric analysis at 3 h post-IRE demonstrated a marked increase in CD45^+^F4/80^+^ macrophages, CD45^+^CD11c^+^ DCs, and CD45^+^CD3^+^CD335^+^ natural killer cells, while CD45^+^Ly6G^+^ neutrophils exhibited only minor changes (Fig. 1C and Fig. S3). This suggests that IRE induces early immune remodeling by promoting the infiltration of innate immune cells into the TME. Transcriptomic profiling at 3 and 9 d after IRE further revealed transient up-regulation of multiple inflammatory and immune-related genes, including Ccl4, Ccl3, Ifng, Tnf, Cd40, Stat1, Il1b, Nos2, Irf7, Il6, Tlr2, Cxcl10, Cxcl9, Irf5, and Ccl5 (Fig. 1D). These gene expression changes indicate the activation of inflammatory signaling and antigen-presentation pathways, suggesting that IRE acts as an immune-priming trigger rather than a purely physical ablation technique.

Collectively, the results in Fig. 1 demonstrate that IRE treatment induces the activation of apoptotic and oxidative-stress pathways, infiltration of innate immune cells, and up-regulation of immune-inflammatory genes, thereby creating a transient window of opportunity for subsequent immuno-nanotherapeutic interventions [9,37,38].

### Characterization and tumor accumulation of NPs after IRE treatment

**Fig. 2.**
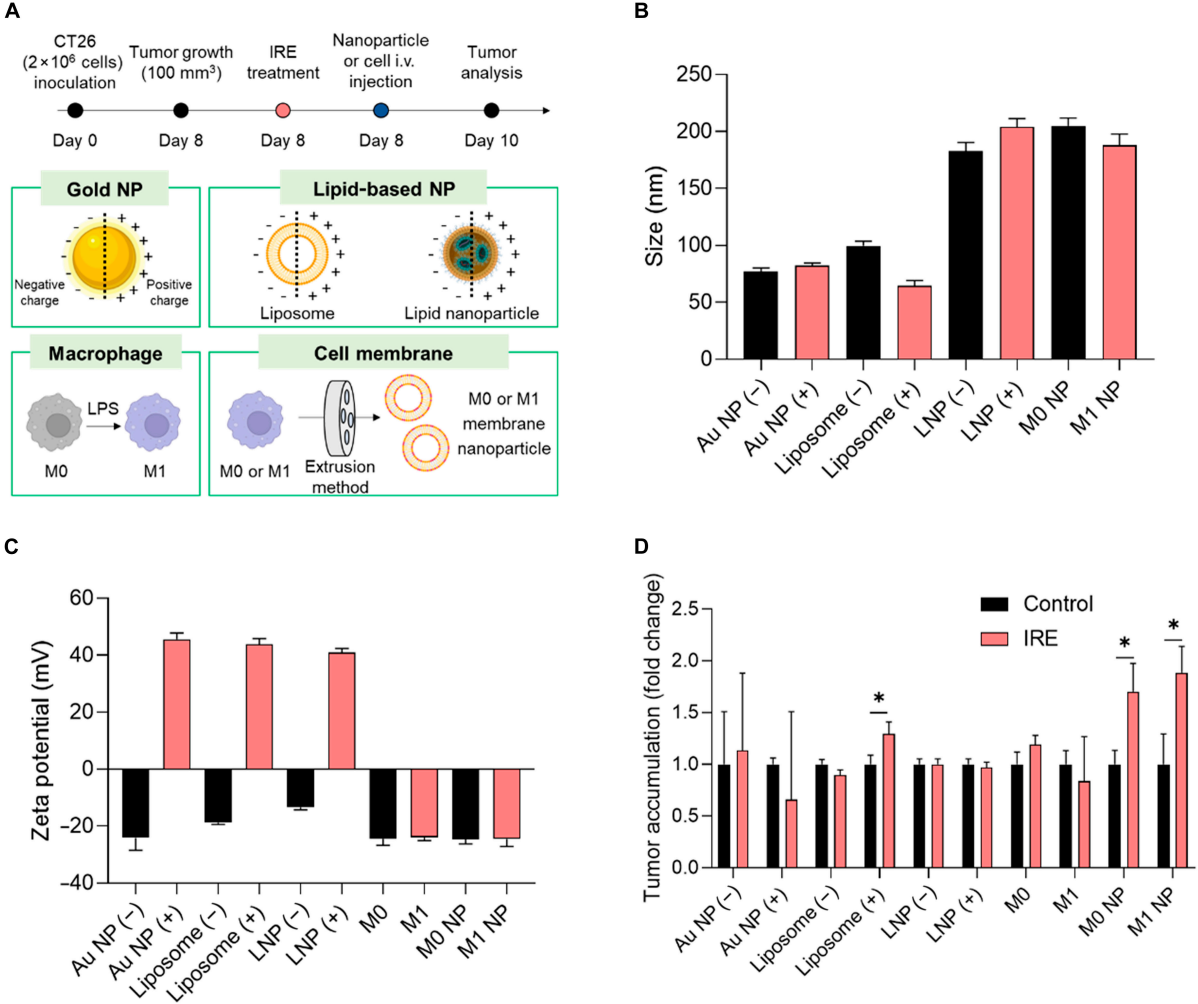
Characterization and tumor accumulation of nanoparticles (NPs) and cell-membrane-based formulations following IRE treatment. (A) Schematic illustration of the experimental design. CT26-tumor-bearing mice were treated with IRE, followed by intravenous injection of various NPs or cell-based formulations, including gold NPs (Au NPs, negatively or positively charged), lipid-based NPs (liposomes or lipid NPs [LNPs], negatively or positively charged), and macrophage (M0 or M1) cell-membrane-derived NPs. LPS, lipopolysaccharide. (B) Hydrodynamic size analysis of each NP formulation based on number-weighted dynamic light scattering distributions. (C) Zeta-potential measurements showing surface charge characteristics of NPs and cell-membrane-derived NPs. (D) Quantification of tumor accumulation of different NP types and cell-membrane-derived NPs in control and IRE-treated tumors (*n* = 3). Data are presented as the mean ± SD (*P < 0.05).

To investigate the effect of IRE treatment on tumor accumulation based on NP surface charge and biomimetic source, various types of NPs were compared, including Au NPs, lipid-based NPs (liposome or LNP), macrophages (M0 or M1), and macrophage-membrane-derived NPs (M0 NPs or M1 NPs) (Fig. 2A). For biodistribution comparison, CT26 tumors were established on both flanks of BALB/c mice, followed by IRE treatment on one side, after which NPs were intravenously administered. This experimental design enabled a direct comparison between IRE-treated and untreated tumors within the same animal, thereby minimizing interindividual biological variability.

Positively charged NPs are known to interact strongly with negatively charged components of the tumor vasculature and extracellular matrix, leading to enhanced tumor retention and cellular uptake compared with negatively charged NPs [39]. To examine the combinatorial potential of this electrostatic property with IRE-induced TME modulation, Au NPs and lipid-based NPs were synthesized in both anionic and cationic forms.

Based on our finding in Fig. 1C that macrophages are the predominant immune population recruited post-IRE, we hypothesized that NPs camouflaged with macrophage membranes would show superior targeting to this remodeled microenvironment. Macrophages are known to exhibit distinct surface ligand profiles depending on their polarization state, which results in differential recognition and adhesion to inflammatory and tumor tissues [40,41]. To further examine how macrophage polarization affects their tumor-homing behavior under IRE-induced inflammatory conditions, both M0 and M1 macrophages and their membrane-derived NPs were prepared and comparatively analyzed.

DLS measurements revealed that only the liposome formulation exhibited a slight size increase, while Au NPs, LNPs, and M0/M1 NPs showed comparable hydrodynamic diameters (Fig. 2B and Fig. S4). These results indicate that surface charge modulation did not markedly affect particle morphology or colloidal stability. Cationic surfaces were achieved by different methods depending on the NP type. Au NPs were coated with PEI, while liposomes and LNPs incorporated DOTAP into their lipid composition (Fig. 2C). These modifications effectively conferred positive zeta potential while maintaining high dispersion stability.

Biodistribution was evaluated by inductively coupled plasma–mass spectrometry for Au NPs and by DiR fluorescence imaging for liposomes, LNPs, and macrophage-derived NPs (Fig. 2D). IRE treatment can increase tumor vascular permeability and thereby facilitate NP infiltration into tumor tissues [42,43]. For Au NPs, charge-dependent differences in biodistribution were observed in the lung and spleen, but these do not influence tumor targeting (Fig. S5). Notably, cationic liposomes showed greater accumulation within IRE-treated tumors than anionic liposomes, consistent with their enhanced electrostatic interaction with negatively charged vascular and extracellular components and improved retention within the TME [16].

In addition, the most pronounced IRE-dependent increase in tumor accumulation was observed for M0 NPs and M1 NPs. The enhanced vascular permeability and concurrent immune cell infiltration induced by IRE may have contributed to improved intratumoral delivery of these NPs. Although these NPs share surface ligands with their parental macrophages [23], their nanoscale dimensions enable them to traverse endothelial gaps and remain longer within the tumor vasculature. Furthermore, M1 NPs show a modest trend toward higher tumor accumulation than M0 NPs after IRE treatment, which may be attributed to the retention of inflammation-responsive adhesion molecules and chemokine receptors on M1 macrophage membranes, promoting stronger interaction with the IRE-induced TME [44].

Collectively, IRE-induced modulation of the TME provides a favorable window for NP delivery. Among the tested formulations, macrophage-membrane-derived NPs demonstrated enhanced tumor accumulation following IRE treatment, supporting the selection of M1 NPs as a suitable delivery vehicle for the combination IRE immunotherapy strategy.

### Synthesis and physicochemical characterization of M1-GAZ nanogels

**Fig. 3.**
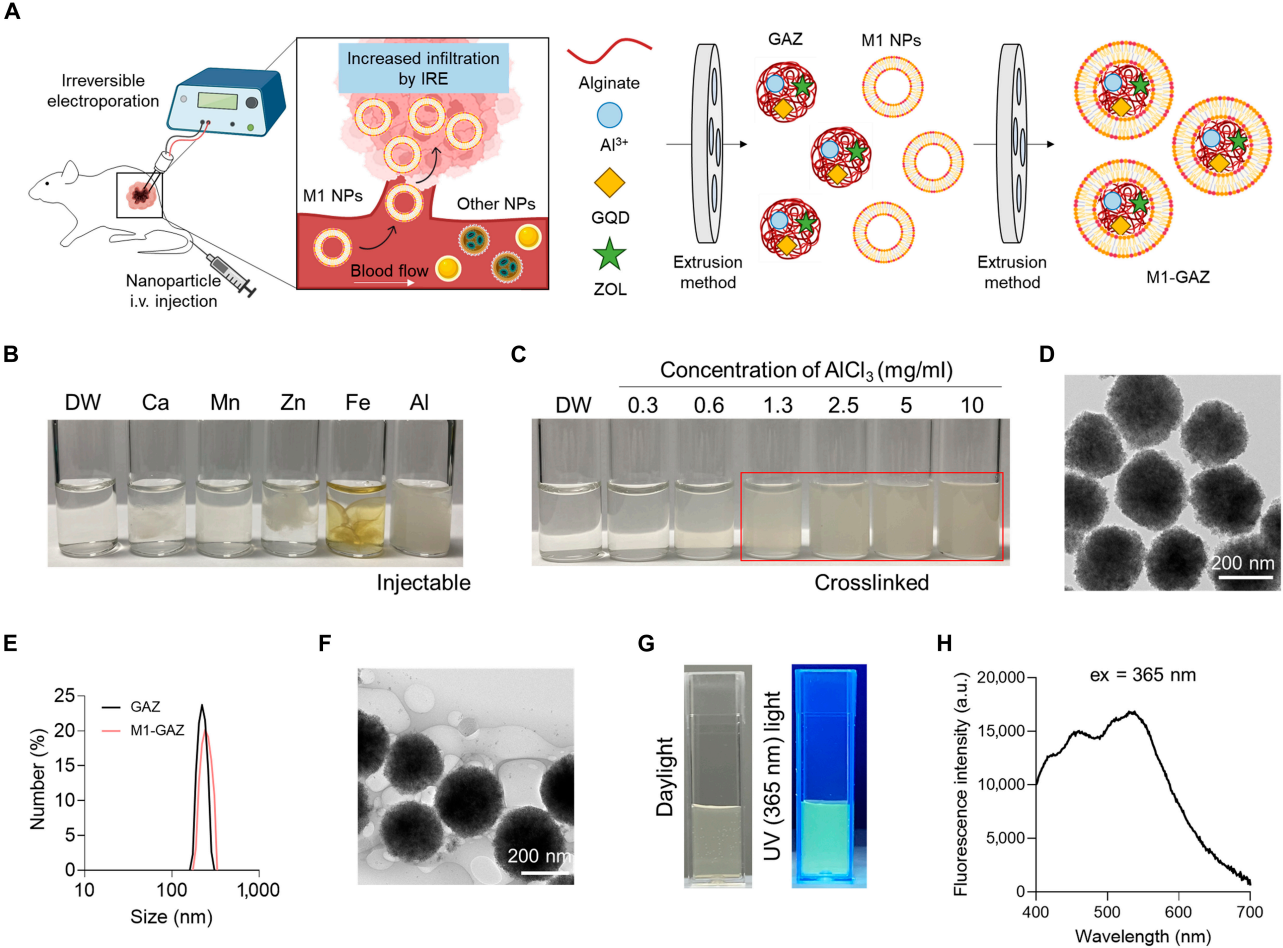
Synthesis and characterization of M1 macrophage-membrane-coated alginate (M1-GAZ) nanogels. (A) Schematic illustration of the design rationale for the M1-GAZ nanogel. IRE enhances infiltration, creating a short delivery window for NP accumulation. To exploit this microenvironment, an alginate-based nanogel was developed and coated with M1 macrophage membranes, incorporating zoledronic acid (ZOL) and graphene quantum dots (GQDs). (B) Screening of ion-mediated alginate crosslinking to identify injectable formulations. Various divalent and trivalent ions (Ca^2+^, Mn^2+^, Zn^2+^, Fe^3+^, and Al^3+^) were tested, and aluminum ions (Al^3+^) produced stable, homogeneous injectable hydrogels. (C) Optimization of AlCl_3_ concentration for alginate crosslinking. Gel formation was observed above 1.3 mg/ml AlCl_3_. (D) Transmission electron microscopy (TEM) image of spherical GAZ nanogels. (E) Hydrodynamic size analysis of GAZ and M1-GAZ based on number-weighted dynamic light scattering distributions. (F) TEM image of spherical M1-GAZ nanogels. (G) Optical appearance of M1-GAZ under daylight and ultraviolet (UV) (365 nm) illumination, exhibiting visible fluorescence. (H) Fluorescence emission spectrum of M1-GAZ at an excitation wavelength of 365 nm.

Based on the previous results that M1 macrophage-derived NPs exhibited the highest tumor accumulation following IRE treatment, we developed an alginate-based nanogel system coated with M1 macrophage membranes (M1-GAZ) to enable synergistic combination therapy with IRE (Fig. 3A). Alginate was selected as the structural matrix owing to its excellent biocompatibility, facile ion-exchange-mediated gelation, suitability for reproducible large-scale fabrication, and stable co-encapsulation capability of multiple therapeutic agents without the need for complex chemical reactions [45,46]. ZOL was incorporated as an immunomodulating small molecule that enhances IRE-induced immune activation. ZOL repolarizes tumor-associated macrophages from the M2 to the M1 phenotype, thereby amplifying local antitumor immunity and complementing the transient immune stimulation elicited by IRE [47,48]. GQDs were introduced as fluorescent nanoprobes to enable real-time visualization and tracking of nanogel biodistribution and tumor accumulation in vivo [49]. Notably, the use of GQDs as fluorescent nanoprobes was intentionally selected over covalent fluorophore conjugation to alginate, as previous studies have shown that quantum-dot-based fluorescent hydrogels can provide strong photostability and signal brightness while preserving the native chemistry and gelation behavior of the polymer network. Such noncovalent incorporation strategies have been widely adopted to enable robust fluorescence without perturbing hydrogel crosslinking or structural integrity [50]. As shown in Fig. S6, the synthesized GQDs exhibited uniform morphology with an average diameter below 5 nm, confirming their nanoscale homogeneity suitable for stable encapsulation within the alginate matrix.

To fabricate nanosized hydrogels suitable for intravenous administration, an extrusion-based approach was employed. To optimize the formation of injectable gels for the extrusion process, the ionic crosslinking efficiency of various multivalent metal ions, including Ca^2+^, Mn^2+^, Zn^2+^, Fe^3+^, and Al^3+^, was systematically evaluated (Fig. 3B). Among these, Al^3+^ ions formed the most uniform and compact gel network, and the gelation efficiency increased proportionally with Al^3+^ concentration (Fig. 3C). Subsequently, ZOL and GQDs were incorporated into the alginate solution, and alginate-based nanogels (GAZ) encapsulating both components were fabricated through Al^3+^-mediated ionic crosslinking followed by extrusion (Fig. 3D). Encapsulation efficiencies of GQDs and ZOL increased with higher AlCl_3_ concentrations and a greater amount of M1 membrane (Fig. S7). Quantification of GQD and ZOL loading was performed based on fluorescence intensity (365-nm excitation) and ultraviolet absorbance measurements, respectively, as shown in Fig. S8A. In addition, fluorescence analysis of the supernatant after purification showed no detectable signal under 365-nm excitation (Fig. S8B), suggesting that GQDs were successfully encapsulated within the M1-GAZ nanogels. However, extrusion became difficult when AlCl_3_ exceeded 10 mg/ml or the membrane amount surpassed 10^12^ particles, indicating these values as the practical upper limits for formulation optimization.

DLS analysis showed that GAZ nanogels had an average hydrodynamic diameter of 223 nm, whereas M1-GAZ nanogels exhibited a slightly larger size of 248 nm, confirming successful membrane coating (Fig. 3E and Fig. S9). Transmission electron microscopy further demonstrated that the spherical morphology and structural integrity of the nanogels were well preserved after M1 membrane coating (Fig. 3F). Thermogravimetric analysis was performed to quantify the membrane content in M1-GAZ (Fig. S10). Thermogravimetric analysis results for the M1-GAZ nanogels indicate that 18 wt.% of the total nanogel mass originated from the M1 membrane coating. Under ultraviolet excitation (365 nm), M1-GAZ nanogels displayed strong fluorescence with a characteristic GQD-derived emission peak (Fig. 3G and H).

Taken together, the M1-GAZ nanogel represents a multifunctional platform that integrates alginate-based structural stability, ZOL-mediated immune modulation, GQD-derived fluorescence, and M1 macrophage-membrane-driven tumor targeting. This synergistic configuration provides a robust and versatile foundation for enhancing immune activation and antitumor efficacy in combinational nano-enabled immunotherapy.

### In vitro cytotoxicity and cytokine secretion profiles of M1-GAZ

**Fig. 4.**
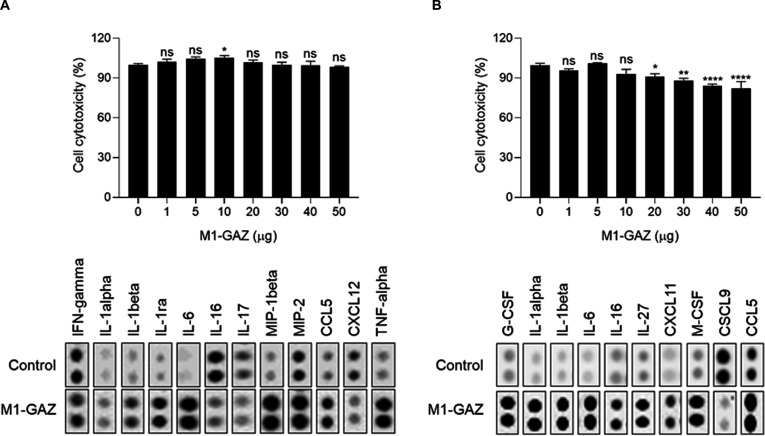
Cell viability and cytokine profiling of M1-GAZ nanogels. (A) (Top) Cell viability of dendritic cells after 24-h incubation with increasing concentrations of M1-GAZ (0 to 50 μg), assessed by Cell Counting Kit-8 (CCK-8) assay (*n* = 3). (Bottom) Corresponding cytokine antibody array of supernatants from DC2.4 cells treated with M1-GAZ (50 μg). (B) (Top) Cell viability of macrophages after 24-h incubation with increasing concentrations of M1-GAZ (0 to 50 μg), assessed by CCK-8 assay (*n* = 3). (Bottom) Corresponding cytokine antibody array of supernatants from RAW264.7 cells treated with M1-GAZ (10 μg). Data are presented as the mean ± SD (*P < 0.05, **P < 0.01, and ****P < 0.0001; ns, not significant).

To investigate the biocompatibility and immunostimulatory potential of the M1-GAZ nanogels, cytotoxicity and cytokine secretion were examined in DCs and macrophages. Cell viability assays revealed that M1-GAZ exhibited negligible cytotoxicity across a wide concentration range (0 to 50 μg/ml) in both DCs and macrophages (Fig. 4A and B, top). Even at the highest dose, cell viability remained above 80%, demonstrating the excellent biocompatibility of the formulation and its suitability for systemic use. To assess the immunomodulatory capability of M1-GAZ, cytokine array analyses were performed (Fig. 4A and B, bottom).

In DCs, treatment with M1-GAZ markedly increased the secretion of pro-inflammatory cytokines and chemokines such as tumor necrosis factor-α, interleukin-6 (IL-6), interleukin-1β (IL-1β), CCL5, CXCL9, and CXCL11 compared to untreated controls (Fig. 4A). Similarly, in macrophages, M1-GAZ induced elevated levels of IL-6, interleukin-1α (IL-1α), macrophage colony-stimulating factor, and granulocyte colony-stimulating factor, indicating the activation of inflammatory signaling pathways (Fig. 4B). Notably, anti-inflammatory cytokines such as interleukin-10 (IL-10) remained largely unchanged, suggesting that M1-GAZ selectively promotes an M1-like pro-inflammatory polarization rather than a global immune activation.

Overall, these findings indicate that M1-GAZ maintains excellent cytocompatibility while effectively enhancing cytokine secretion and immune activation in both DCs and macrophages, further supporting its role in enhancing IRE-induced immune activation as a nano-immunomodulatory platform for immunotherapy.

### In vivo biodistribution and therapeutic efficacy of M1-GAZ combined with IRE treatment

**Fig. 5.**
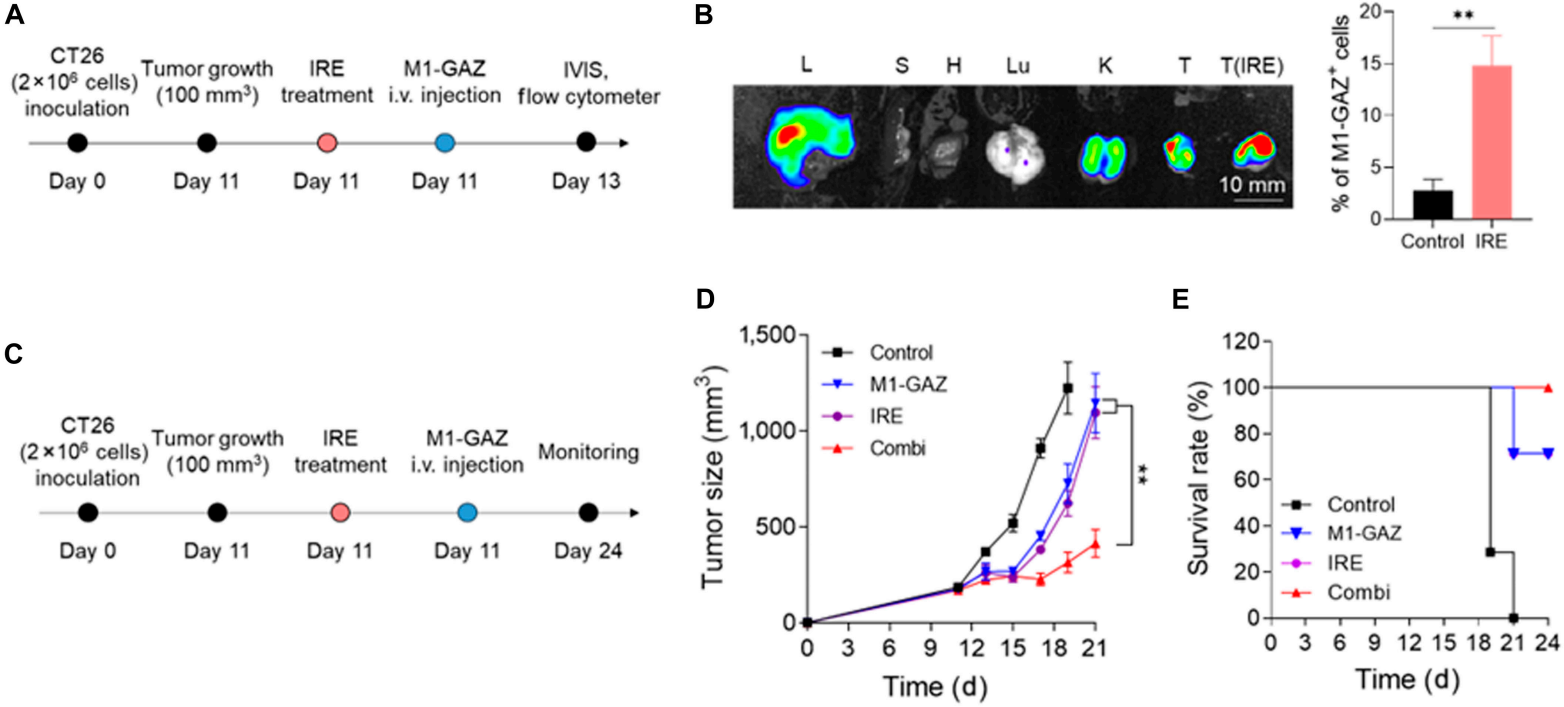
In vivo biodistribution and therapeutic efficacy of M1-GAZ nanogels combined with IRE in CT26-tumor-bearing mice. (A) Schematic illustration of the experimental schedule for evaluating M1-GAZ biodistribution after IRE treatment. CT26-tumor-bearing mice received IRE followed by intravenous injection of M1-GAZ. (B) Biodistribution of M1-GAZ visualized by an in vivo imaging system (IVIS) in major organs [L: liver, S: spleen, H: heart, Lu: lung, K: kidney, T: tumor, and T(IRE): IRE-treated tumor]. Flow cytometry quantification showed significantly enhanced tumor accumulation of M1-GAZ-positive cells in IRE-treated tumors compared with control tumors (*n* = 3, **P < 0.01). (C) Experimental timeline for assessing the therapeutic efficacy of IRE and M1-GAZ combination treatment. (D) Tumor growth curves demonstrating marked inhibition of tumor progression in the combination (IRE + M1-GAZ) group (*n* = 8, **P < 0.01). (E) Kaplan–Meier survival analysis showing prolonged survival in the combination-treated mice (*n* = 8).

To elucidate the role of IRE in promoting M1-GAZ accumulation, a bilateral CT26 tumor model was established on both flanks of BALB/c mice. IRE treatment was applied to one side only, followed by intravenous injection of M1-GAZ nanogels (Fig. 5A).

Since M1-GAZ was intrinsically labeled with GQDs, biodistribution and tumor uptake could be visualized using an in vivo fluorescence imaging system and quantified for cellular association by flow cytometry analysis. Two days after intravenous administration, IVIS imaging revealed that M1-GAZ primarily accumulated in the liver and kidneys, with minimal presence in other organs. Notably, fluorescence intensity was markedly higher in IRE-treated tumors compared with untreated counterparts (Fig. 5B). Consistently, flow cytometry analysis demonstrated an approximately 5-fold higher M1-GAZ signal (representing cellular uptake and association) in the IRE-treated tumors. These findings are in line with the results shown in Fig. 2, confirming that IRE not only enhances the accumulation of M1 NPs but also facilitates the efficient intratumoral delivery of M1 membrane-coated nanogels such as M1-GAZ.

To evaluate the therapeutic efficacy, the CT26 tumor model received IRE treatment followed by intravenous M1-GAZ administration. Tumor growth was monitored until the average tumor volume in the control group reached approximately 1,500 mm^3^ (Fig. 5C). As shown in Fig. 5D, the combination of IRE and M1-GAZ significantly suppressed tumor growth compared with either monotherapy group. Moreover, survival analysis revealed that the combination group exhibited a markedly prolonged median survival and the highest overall survival rate among all experimental groups (Fig. 5E).

Altogether, these results demonstrate that IRE treatment enhanced both the intratumoral accumulation and therapeutic efficacy of M1-GAZ. The synergistic interaction between IRE-induced TME remodeling and M1 macrophage-membrane-based nanotherapy results in potent tumor suppression, underscoring M1-GAZ as a promising IRE-assisted nanotherapeutic platform.

### In vivo intratumoral immune activation efficacy of M1-GAZ combined with IRE treatment

**Fig. 6.**
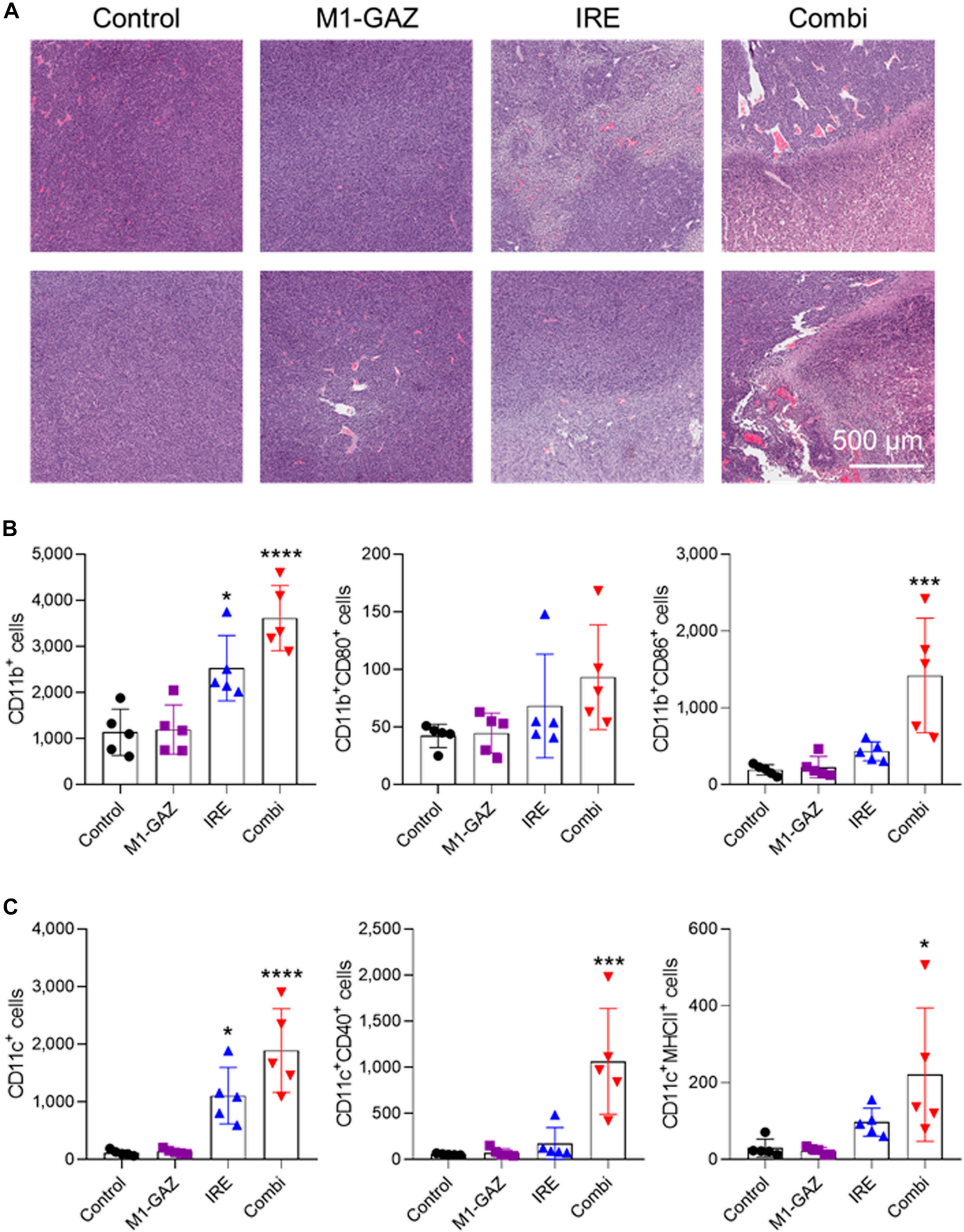
Histological and immune profiling of tumors after IRE and M1-GAZ combination treatment. (A) Representative hematoxylin-and-eosin-stained tumor sections from control, M1-GAZ, IRE, and combination (IRE + M1-GAZ) groups. (B) Quantification of tumor-infiltrating macrophage populations by flow cytometry, including CD11b^+^, CD11b^+^CD80^+^, and CD11b^+^CD86^+^ cells (*n* = 5). (C) Analysis of dendritic cell populations in tumors, including CD11c^+^, CD11c^+^CD40^+^, and CD11c^+^MHCII^+^ cells (*n* = 5). Data are presented as the mean ± SD (*P < 0.05, ***P < 0.001, and ****P < 0.0001).

To comprehensively evaluate the intratumoral immune activation and systemic biocompatibility induced by the combination of M1-GAZ and IRE therapy, histological, immunological, and biochemical analyses were conducted 3 d after treatment. Hematoxylin and eosin staining revealed distinct histopathological features among groups (Fig. 6A). In the control group, the tumor exhibited high cellular density with minimal necrosis or immune cell infiltration was observed. The M1-GAZ monotherapy group showed mild perivascular immune cell infiltration, while the overall tumor architecture remained intact. In contrast, the IRE-treated group exhibited evident tumor necrosis with peripheral immune cell infiltration, consistent with IRE-induced tumor cell death. Notably, the combination group displayed extensive tumor necrosis accompanied by dense infiltration of immune cells, indicating that IRE-mediated tumor ablation synergized with the immunomodulatory activity of M1-GAZ to induce potent immunogenic tumor destruction.

Flow cytometry analysis was performed to quantitatively assess immune cell activation within the TME (Fig. 6B and C and Fig. S11). In macrophages, the combination group showed a trend toward elevated CD80^+^ expression and significantly increased CD86^+^ expression (both M1-associated markers), confirming polarization toward a pro-inflammatory, antitumor phenotype. This effect is attributed to the synergistic interaction between IRE-induced tumor antigen release and likely the sustained release of ZOL from the nanogels, which drives potent immunometabolism reprogramming within M1-GAZ. Similarly, DC analysis revealed a significant up-regulation of activation marker CD40^+^ and a more modest increase in MHCII^+^, suggesting enhanced antigen presentation and adaptive immune activation. This enhanced DC activation is likely a result of a synergistic crosstalk. IRE-released antigens prime the DCs, and the M1-GAZ-induced pro-inflammatory TME driven by both the nanogel itself and the repolarized M1 macrophages provide the costimulatory signals required for full DC maturation.

Systemic biosafety evaluation showed that all hematological parameters remained within normal physiological ranges, and no notable alterations were observed in hepatic or renal function indicators (Fig. S12). Furthermore, hematoxylin and eosin staining of major organs (heart, lung, liver, spleen, and kidney) showed no evidence of tissue damage, confirming the absence of systemic toxicity (Fig. S13).

These results demonstrate that the IRE combined with M1-GAZ induces robust intratumoral immune activation and immunogenic cell death while maintaining excellent systemic biocompatibility. The synergistic interplay between IRE-mediated immunogenic modulation and M1-GAZ-driven immune regulation establishes an effective and safe nanotherapeutic strategy that simultaneously achieves potent local immune activation and systemic safety, representing a promising platform for IRE-assisted tumor-targeted immunotherapy.

## Conclusion

In this study, we developed an alginate-based nanogel coated with M1 macrophage membranes (M1-GAZ) and demonstrated its synergistic therapeutic efficacy when combined with IRE. Comprehensive time-resolved analyses revealed that IRE rapidly activates apoptotic and oxidative-stress pathways, increases vascular permeability, and promotes early recruitment of innate immune cells, thereby creating a transient but highly permissive TME for improved delivery of M1-GAZ.

To effectively exploit the limited temporal window created by IRE-induced TME priming, we adopted a delivery strategy that is minimally procedure dependent and compatible with systemic intravenous administration. Based on this rationale, various NP formulations differing in surface charge and biomimetic origin were compared to identify the most suitable delivery platform within the IRE-modulated TME. Among all NP types examined, M1 NPs showed the greatest tumor accumulation following IRE, indicating their preferential interaction with the IRE-induced inflammatory and immunologically remodeled microenvironment and confirming their suitability for enhanced immune-based therapy.

Based on these observations, we rationally designed M1-GAZ as a multifunctional nanoplatform that integrates the biocompatible and ionically crosslinkable properties of alginate, the immune-metabolic reprogramming capacity of ZOL, the fluorescence traceability of GQDs, and M1 macrophage membrane coating for improved tumor targeting after IRE-mediated TME priming.

In vitro, M1-GAZ exhibited excellent biocompatibility while stimulating robust secretion of pro-inflammatory cytokines in both macrophages and DCs. In vivo, the combination of IRE and M1-GAZ significantly improved intratumoral nanogel accumulation, promoted immunogenic tumor cell death, and markedly increased activation of macrophages and DCs. These coordinated immune responses effectively converted a “cold” tumor into a “hot” one, resulting in significant tumor growth inhibition and prolonged overall survival. Hematological and histopathological evaluations revealed no structural abnormalities or systemic toxicity in major organs, confirming the excellent biocompatibility of this therapeutic approach.

These findings demonstrate that the synergistic interplay between IRE-induced TME priming and M1-GAZ-mediated immune regulation effectively reprograms the TME toward an active immune phenotype while maintaining systemic safety. Therefore, the IRE-assisted M1 macrophage-membrane-based nanoplatform represents a highly effective and biocompatible strategy for achieving localized immune activation and durable antitumor immunity in biomimetic, nano-enabled cancer immunotherapy. This strategy of using biomimetic nanogels to “hijack” and “amplify” transient, ablation-induced inflammation represents a powerful and potentially generalizable platform for next-generation combination cancer immunotherapies.

## Ethical Approval

All in vivo experiments were conducted under the guidelines of an approved protocol from the Institutional Animal Care and Use Committee of the Sungkyunkwan University of Korea (Republic of Korea, IACUC-2021-017-01).

## Data Availability

The data that support the findings of this study are available on request from the corresponding authors.
