# Supplementary material for: Biomimetic Nanogels Programmed for Irreversible-Electroporation-Primed Tumor Microenvironments to Elicit Durable Antitumor Immunity
Source: Biomater Res. 2026 Jun 25;30:0359. doi: 10.34133/bmr.0359 (PMC13294544; doi:10.34133/bmr.0359)
Supplement: Supplementary 1 — Figs. S1 to S13 [file bmr.0359.f1.docx]

**Supplementary material**

**Title**

**Biomimetic Nanogels Programmed for Irreversible Electroporation-Primed Tumor Microenvironments to Elicit Durable Anti-Tumor Immunity**

**Authors**

Jun-Hyeok Han,^1†^ Ha Eun Shin,^2^ Chun Gwon Park,^3,4^ Hyun-Do Jung,^5^ Jung-Hoon Park,^6,7^
Ji Hoon Jeong,^8,9,10^ Yong Taik Lim,^11^ Dong-Hyun Kim,^1,12,13*^ and Wooram Park^2,9*^

**Affiliations**

^1^Department of Radiology, Feinberg School of Medicine, Northwestern University, Chicago, IL 60611, USA

^2^Department of Integrative Biotechnology, College of Biotechnology and Bioengineering, Sungkyunkwan University (SKKU), Suwon, Gyeonggi 16419, Republic of Korea

^3^Department of Biomedical Engineering, Institute for Cross-disciplinary Studies (ICS), SKKU, Suwon, Gyeonggi 16419, Republic of Korea

^4^Department of Intelligent Precision Healthcare Convergence, ICS, SKKU, Suwon, Gyeonggi 16419, Republic of Korea

^5^Division of Materials Science and Engineering, Hanyang University, Seoul 04763, Republic of Korea

^6^Department of Convergence Medicine, Asan Medical Center, University of Ulsan College of Medicine, Seoul 05505, Republic of Korea

^7^Biomedical Engineering Research Center, Asan Institute for Life Sciences, Asan Medical Center, Seoul 05505, Republic of Korea

^8^School of Pharmacy, SKKU, Suwon, Gyeonggi 16419, Republic of Korea

^9^Department of MetaBioHealth, School of Medicine, SKKU, Suwon, Gyeonggi 16419, Republic of Korea

^10^Biomedical Institute for Convergence at SKKU, SKKU, Suwon, Gyeonggi 16419, Republic of Korea

^11^SKKU Advanced Institute of Nanotechnology (SAINT), Department of Nano Science and Technology, Department of Nano Engineering, and School of Chemical Engineering, SKKU, Suwon, Gyeonggi 16419, Republic of Korea

^12^Department of Biomedical Engineering, McCormick School of Engineering, Evanston, IL 60208, USA

^13^Robert H. Lurie Comprehensive Cancer Center, Chicago, IL 60611, USA

* Address correspondence to: Prof. Wooram Park (E-mail: parkwr@skku.edu), Prof. Dong-Hyun Kim (E-mail: dhkim@northwestern.edu)

**
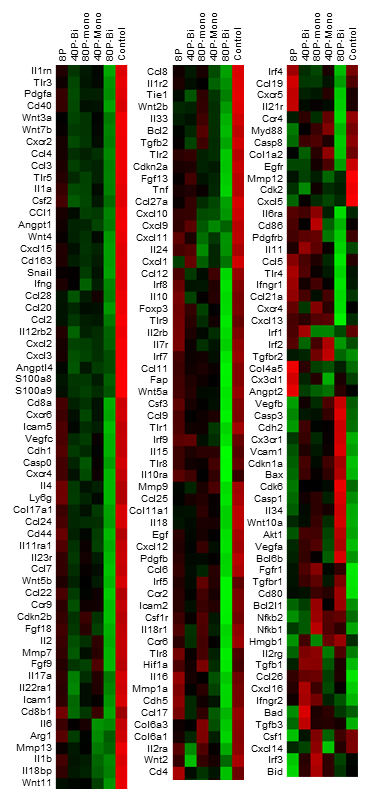
**

**Figure S1. Transcriptomic profiling of tumors following different irreversible electroporation (IRE) pulse conditions.** IRE was performed with a voltage of 1000 V, a pulse duration of 100 µs, and an electrode spacing of 5 mm, applying 8, 40 (mono or bipolar), 80 (mono or bipolar) pulses to evaluate pulse-dependent transcriptomic alterations.

**
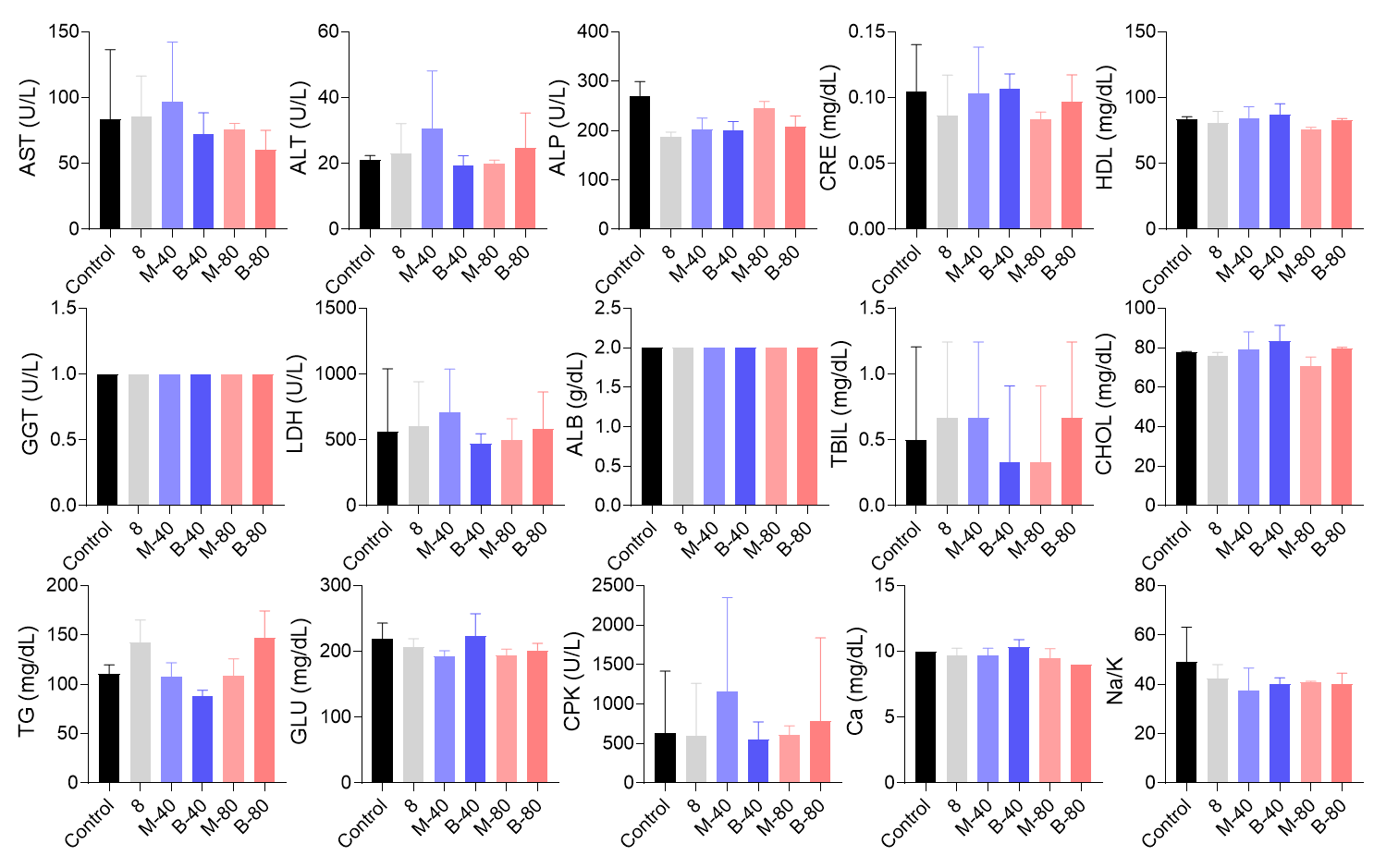
**

**Figure S2. Blood biochemical analysis after irreversible electroporation (IRE) treatment.** Serum levels of key biochemical indicators, including alanine aminotransferase (ALT), aspartate aminotransferase (AST), and creatine phosphokinase (CPK), were measured to assess hepatic, renal, and cardiac function after IRE treatment.

**
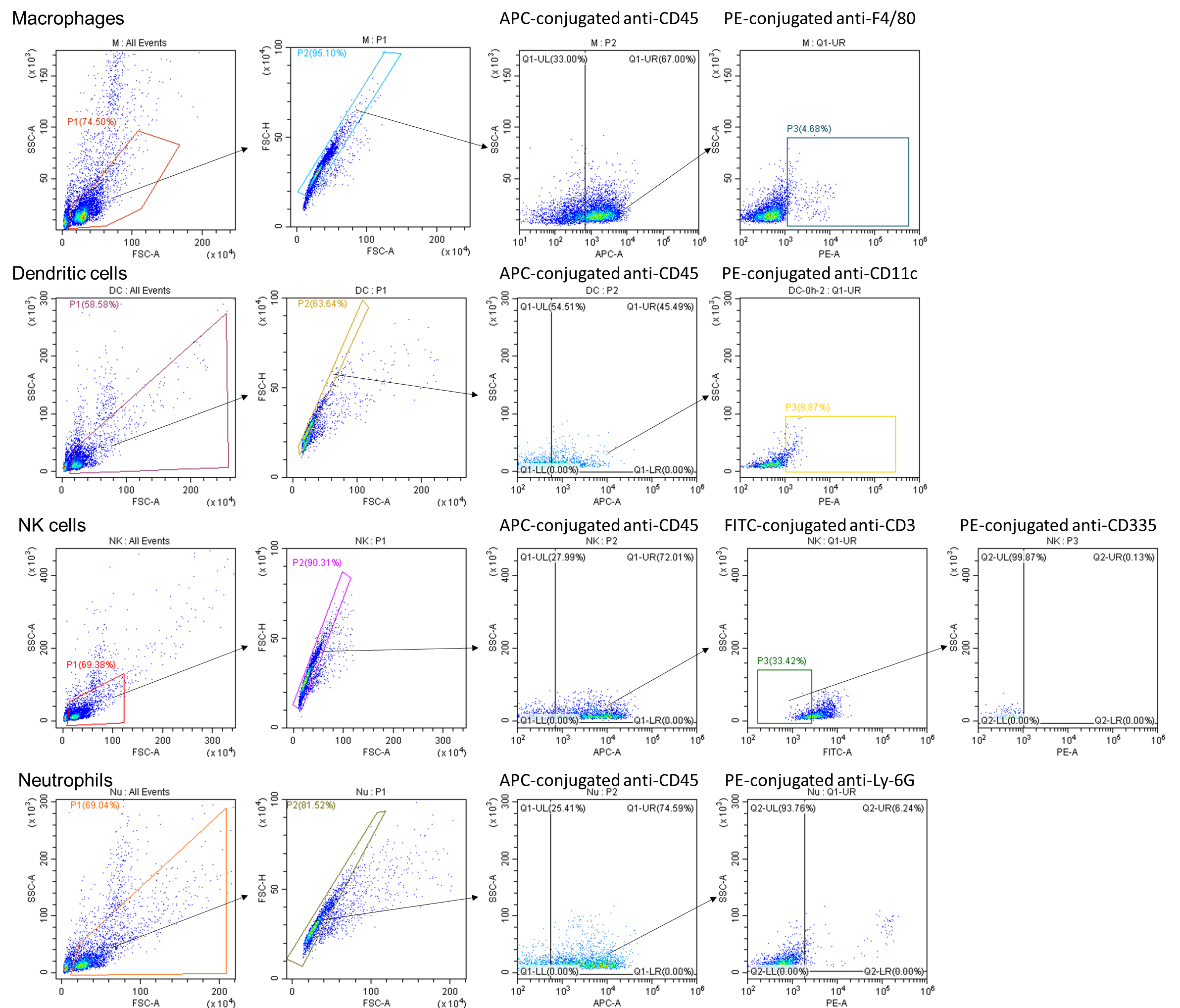
**

**Figure S3. Flow cytometry gating strategy for immune cell populations 3 h after irreversible electroporation (IRE) treatment.** Flow cytometry gating strategy used to identify macrophages (CD45^+^F4/80^+^), dendritic cells (CD45^+^CD11c^+^), NK cells (CD45^+^CD3^-^CD335^+^), and neutrophils (CD45^+^Ly-6G^+^).

.

**
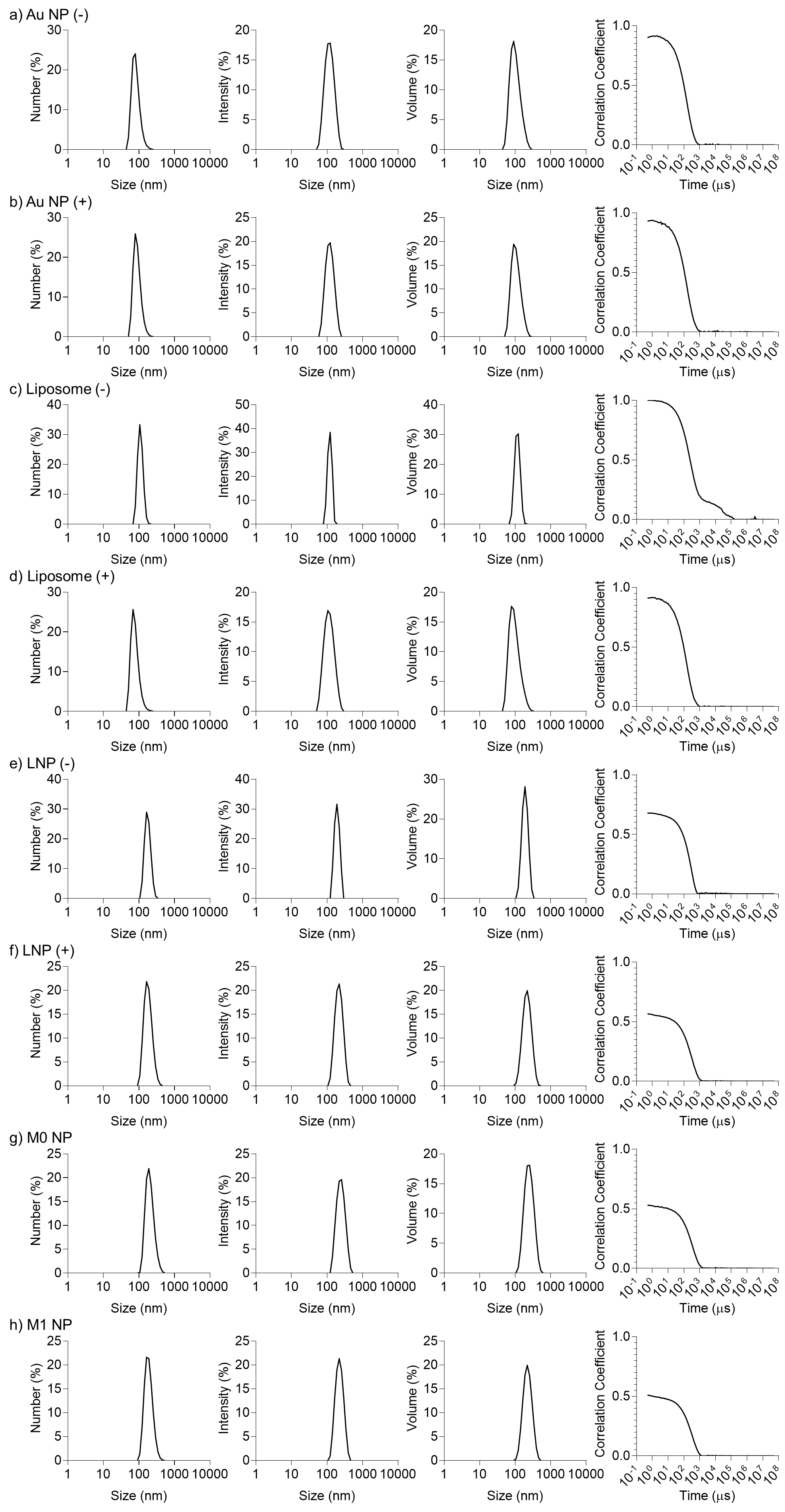
**

**Figure S4. Dynamic light scattering (DLS) measured hydrodynamic sizes of nanoparticle formulations.** The hydrodynamic diameters of (a, b) gold nanoparticles (Au NPs, negatively or positively charged), (c, d, e, f) lipid-based nanoparticles (liposomes or lipid NPs, negatively or positively charged), and (g, h) macrophage (M0 or M1) cell membrane–derived nanoparticles were measured by DLS.

**Figure S5. Biodistribution of Au nanoparticles (Au NPs) after irreversible electroporation (IRE) treatment.** Quantitative analysis of AuNP accumulation in major organs (H: heart, Lu: lung, Li: liver, K: kidney, S: spleen, T: tumor, T (IRE): IRE treated tumor) of CT26 tumor-bearing mice after intravenous injection. Both negatively (Au NP–) and positively (Au NP+) charged NPs were administered following IRE treatment. Au content was measured by ICP-MS and normalized to organ weight.

**
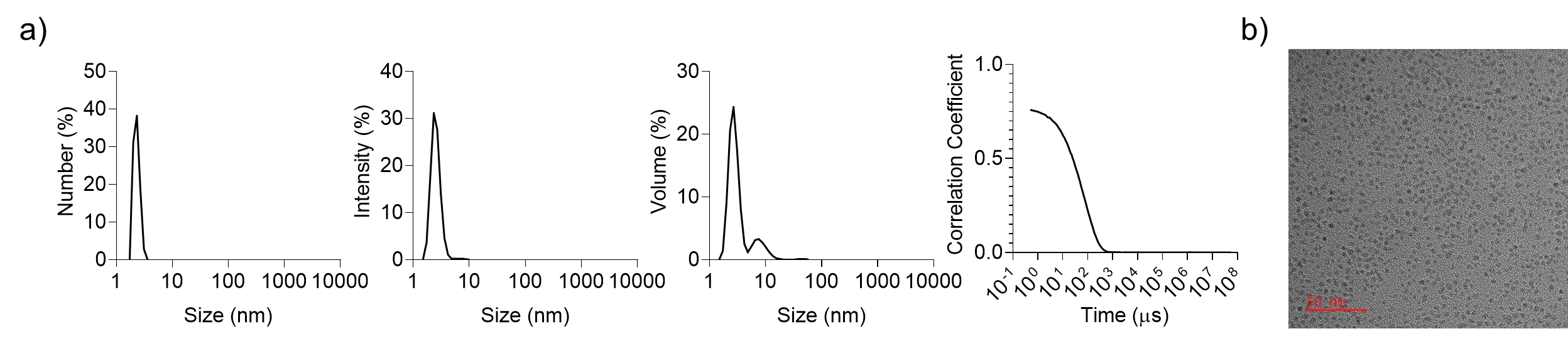
**

**Figure S6. Morphological and size characterization of synthesized graphene quantum dots (GQDs).** Dynamic light scattering analysis (a) and transmission electron microscopy image (b) of GQDs.

**
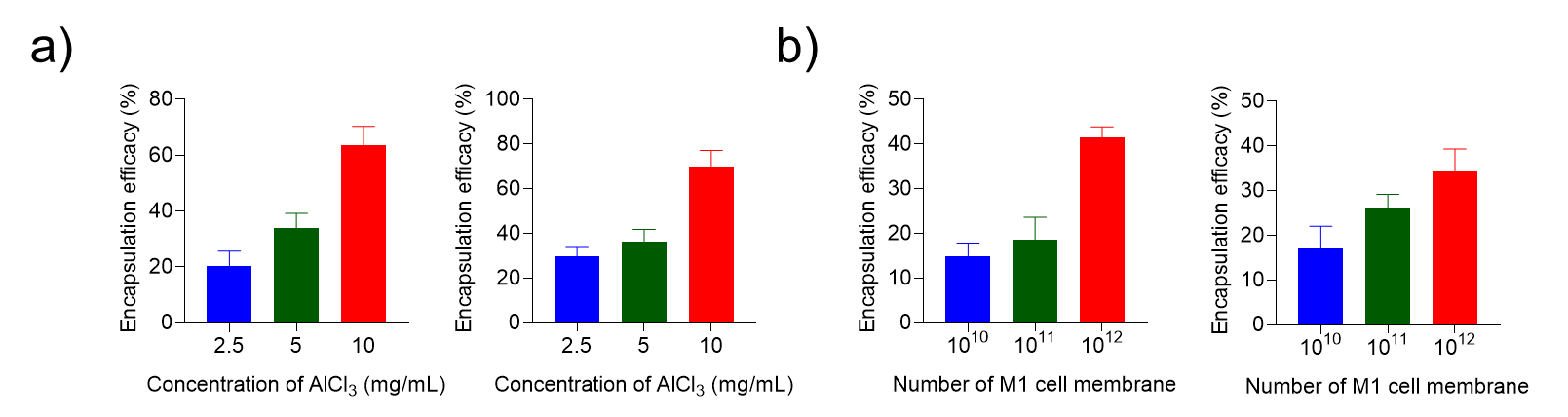
**

**Figure S7. Optimization of encapsulation efficiency of graphene quantum dots (GQDs) and zoledronic acid (ZOL).** (a) Encapsulation efficiency of GQD (left) and ZOL (right) in GAZ nanogels as a function of AlCl_3_ concentration. (b) Encapsulation efficiency of GQD (left) and ZOL (right) in M1-GAZ nanogels as a function of the M1 cell membrane amount (particle number).

**
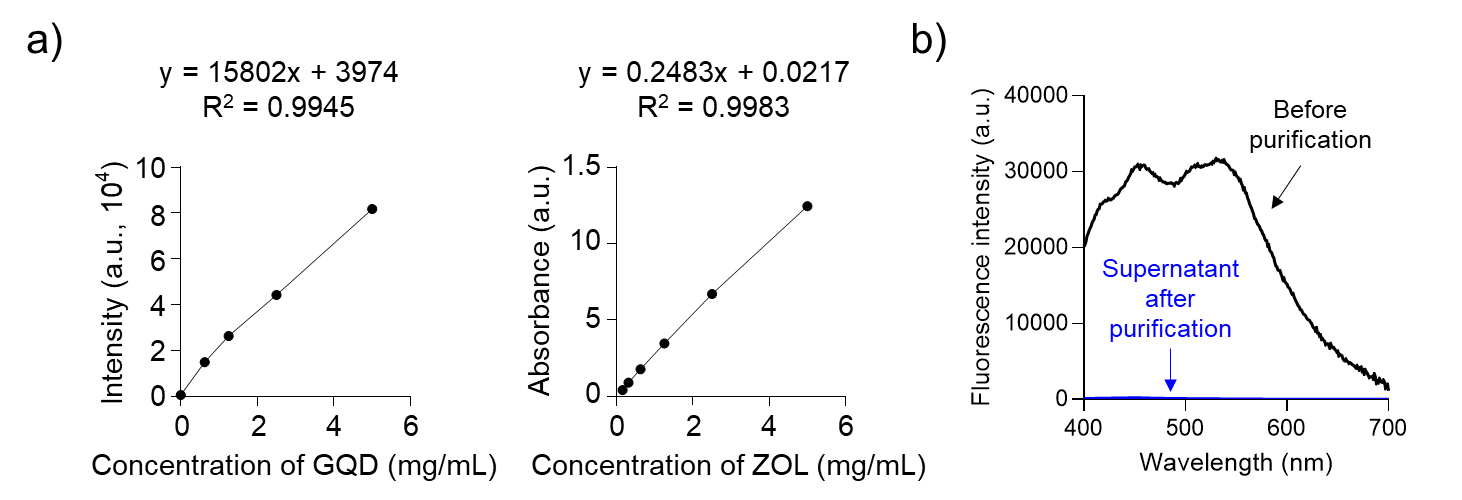
**

**Figure S8. Calibration curves for quantum dots (GQDs) and zoledronic acid (ZOL) and fluorescence analysis of supernatant before and after purification.** (a) The standard curve of GQDs was generated based on fluorescence intensity under 365/530 nm ex/em, while the standard curve of ZOL was obtained by measuring UV absorbance at 230 nm. (b) Fluorescence spectra of the M1-GAZ suspension before purification and the corresponding supernatant after purification, measured under 365 nm excitation.

**
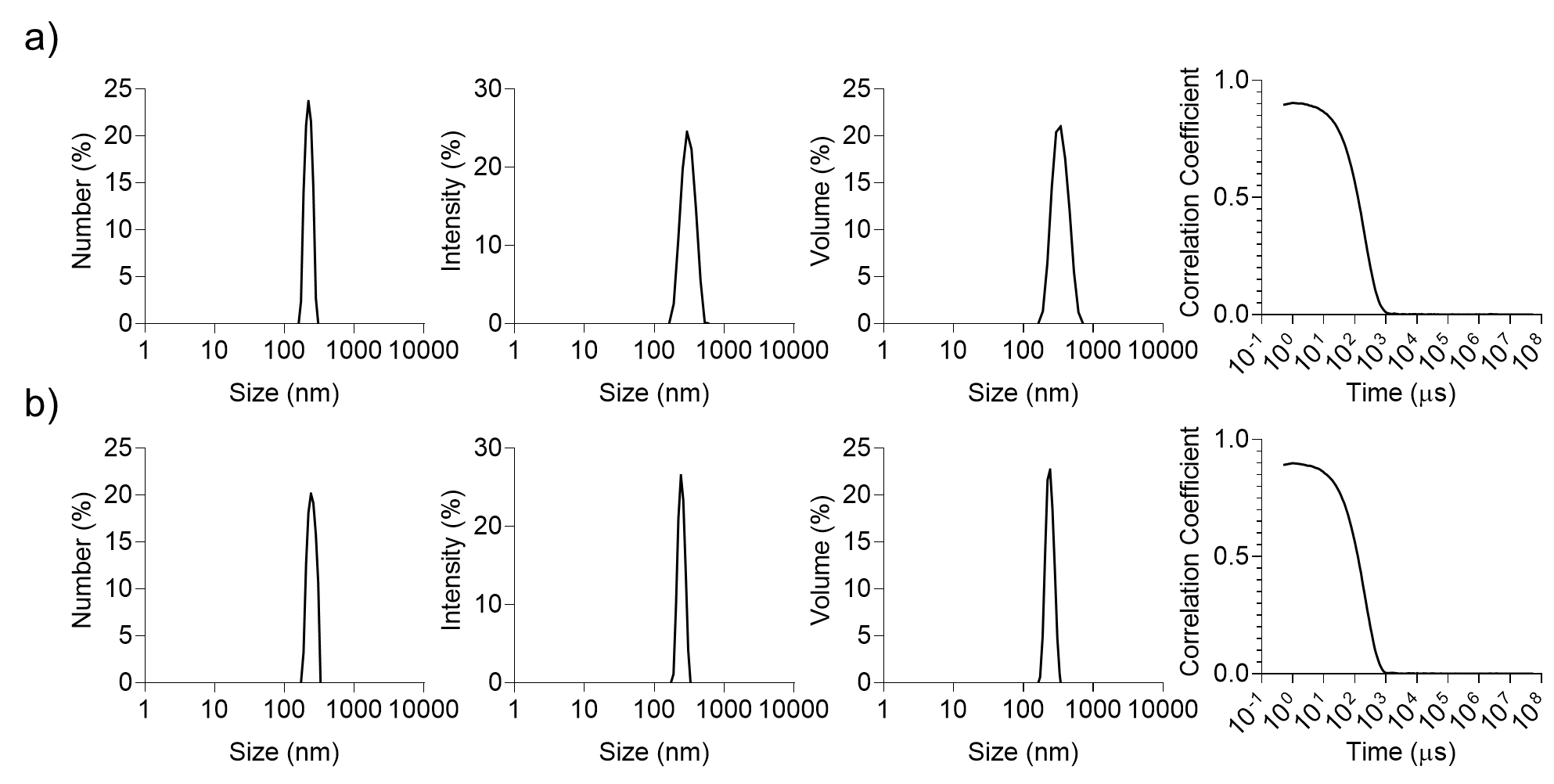
**

**Figure S9. Dynamic light scattering measured hydrodynamic sizes of GAZ and M1-GAZ.**

**Figure S10. Thermogravimetric analysis (TGA) of GAZ, and M1-GAZ formulations.** TGA curves showing the thermal degradation profiles of GQD/ZOL-loaded alginate nanogel (GAZ), and M1 cell membrane-coated GAZ (M1-GAZ).

**
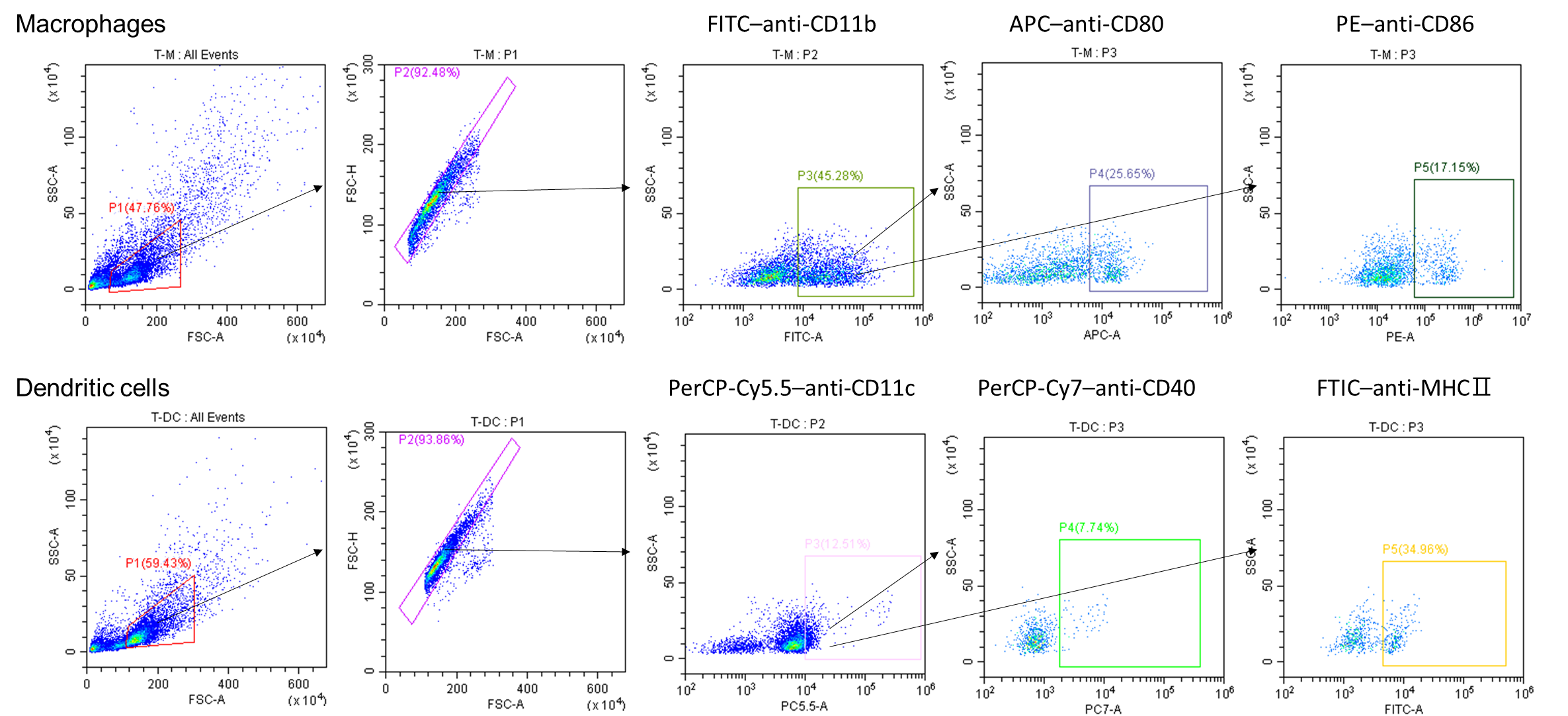
**

**Figure S11. Flow cytometry gating strategy for immune cell populations 3 days after treatment with M1-GAZ nanogels and irreversible electroporation (IRE).** Flow cytometry gating strategy used to identify macrophages (CD11b^+^, CD11b^+^CD80^+^ and CD11b^+^CD86^+^), dendritic cells (CD11c^+^, CD11c^+^CD40^+^, CD11c^+^MHCⅡ^+^).

**
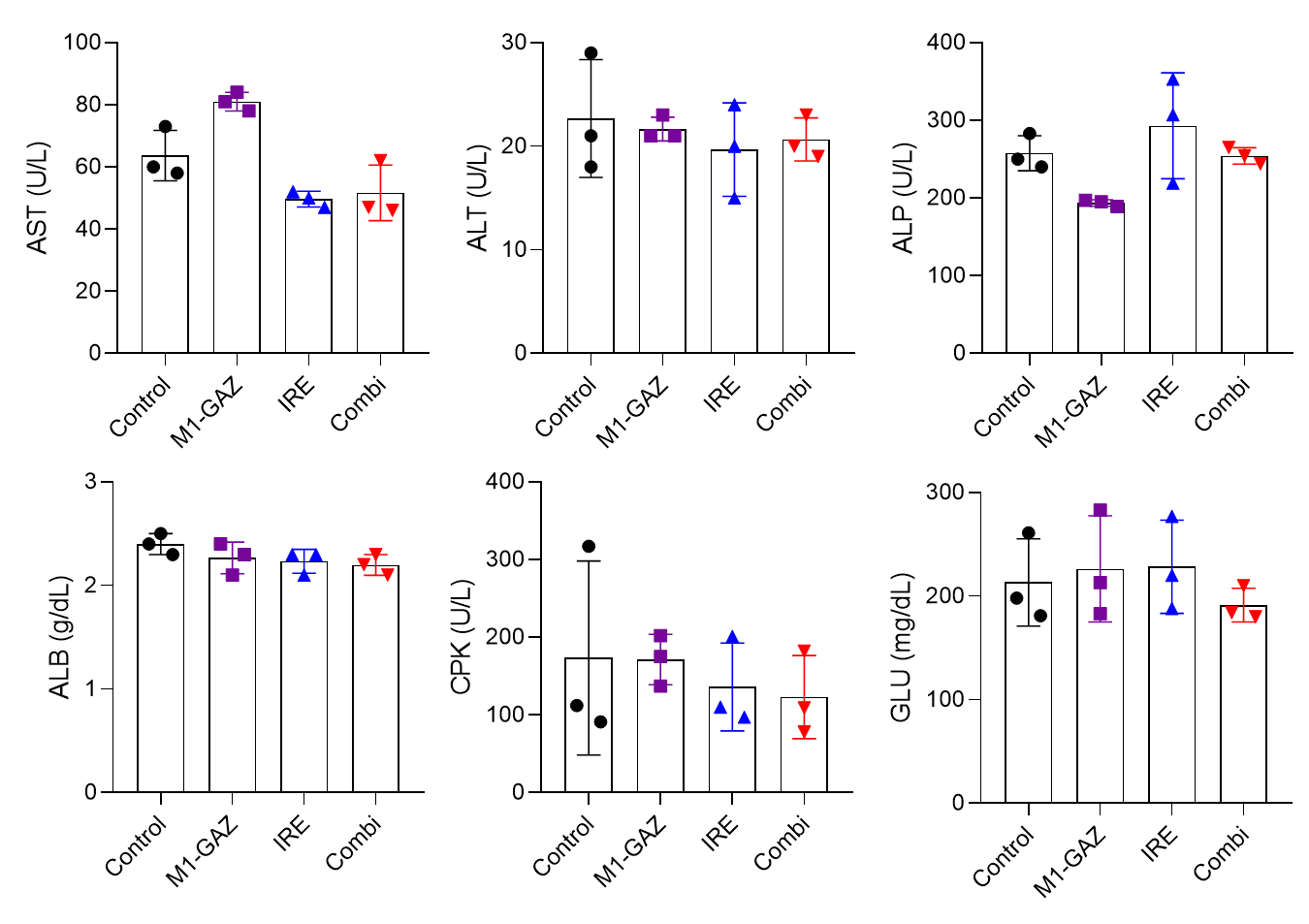
**

**Figure S12. Blood biochemical analysis after combination treatment with M1-GAZ nanogels and irreversible electroporation (IRE).**


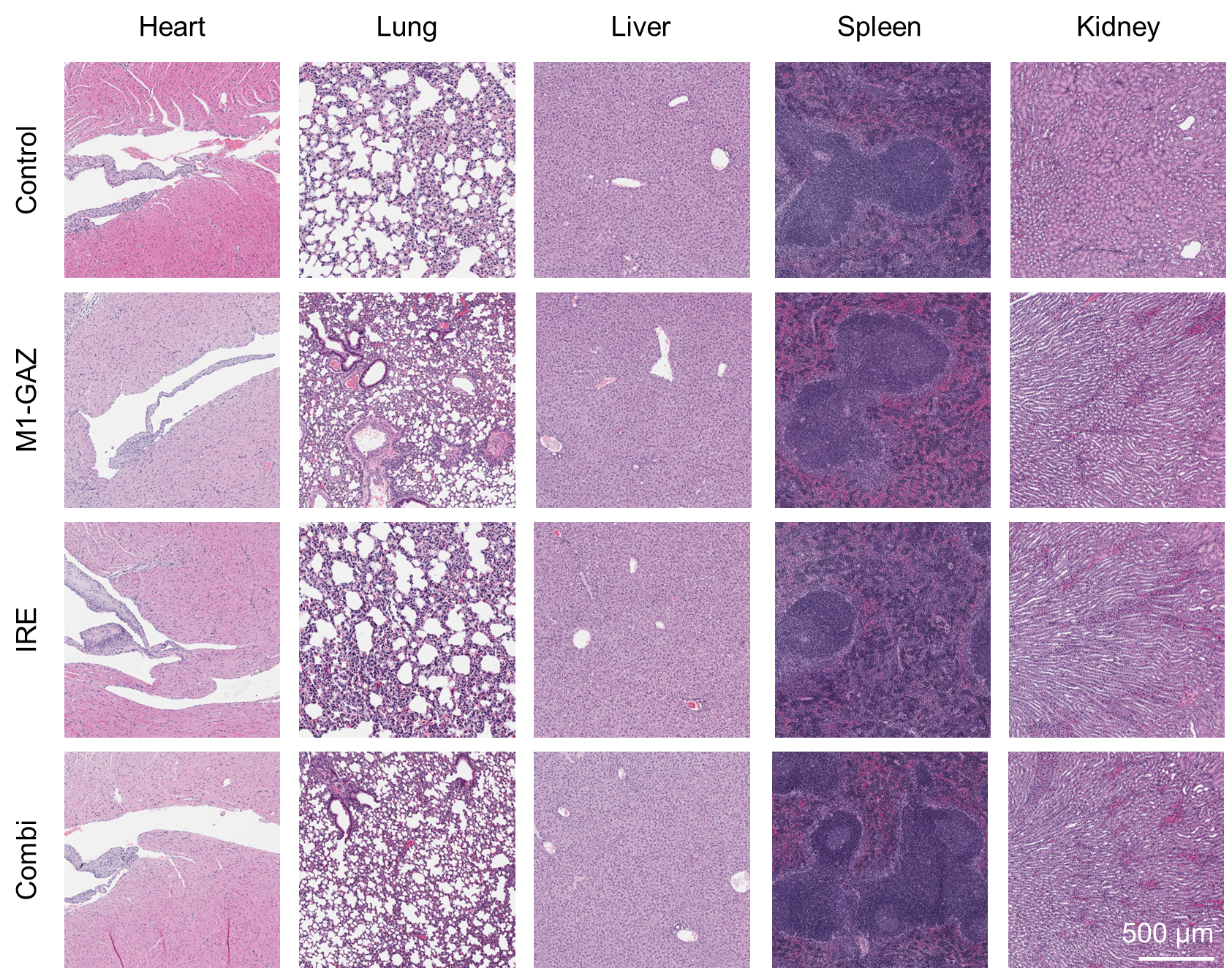


**Figure S13. Histopathological evaluation of major organs after irreversible electroporation (IRE) and M1-GAZ treatment.**
